# Supplementary figures and images for: Sodium azide mutagenesis induces a unique pattern of mutations
Source: PLoS Genet. 2025 Jun 3;21(6):e1011634. doi: 10.1371/journal.pgen.1011634 (PMC12162104; doi:10.1371/journal.pgen.1011634)

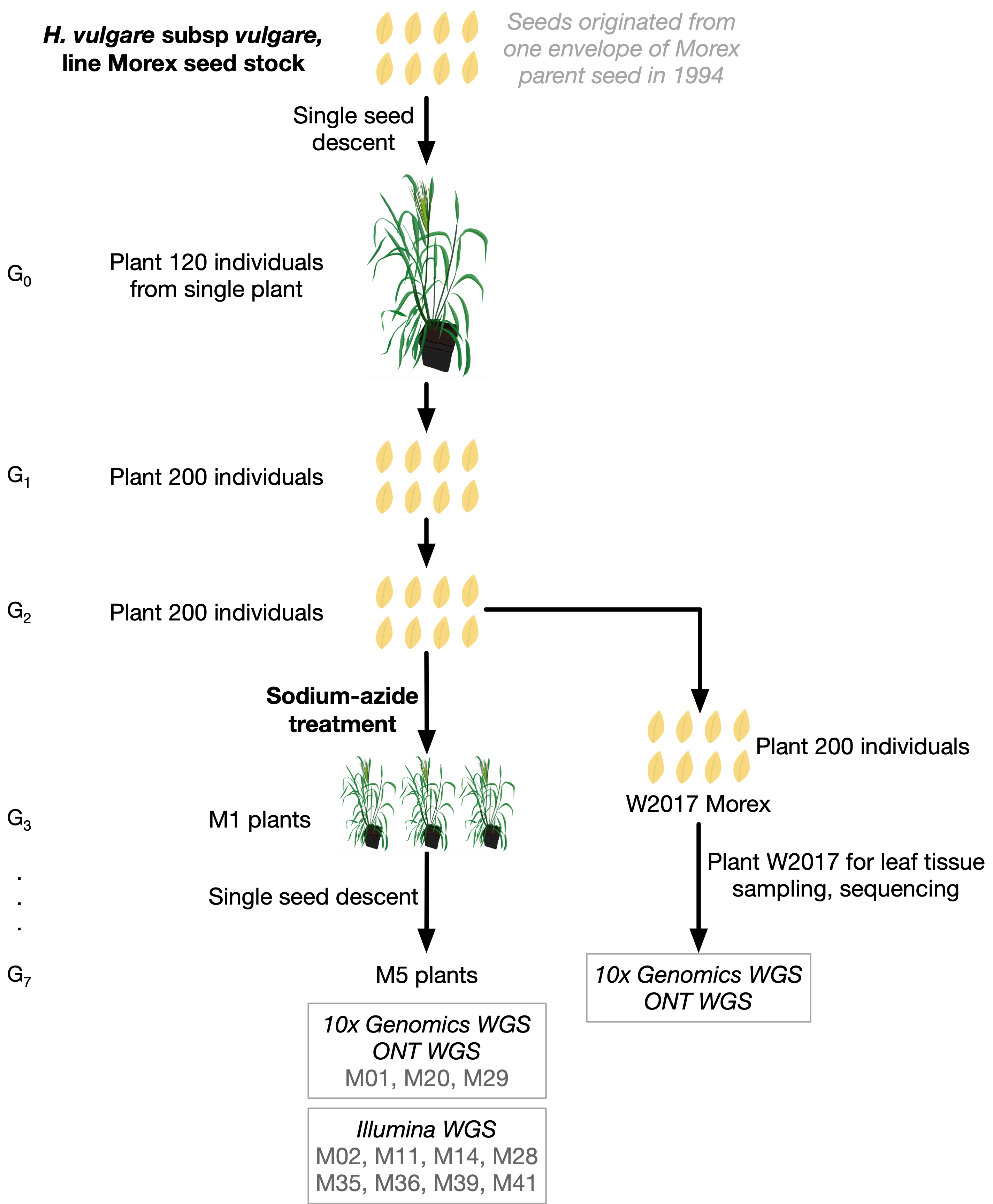

Supplement: S1 Fig — Gx is the generation used for spontaneous mutation estimates (see Methods). (TIFF) [file pgen.1011634.s001.tiff]

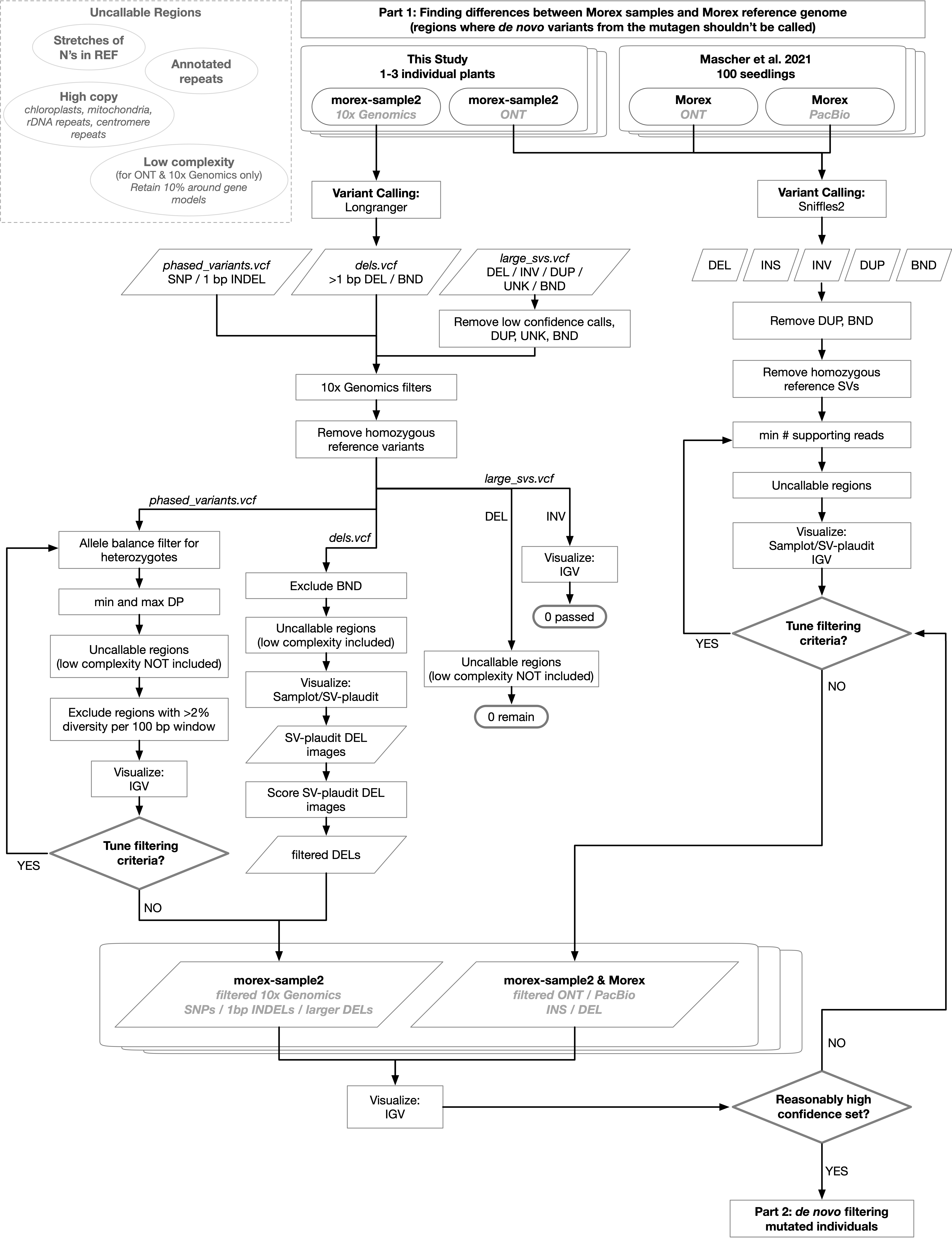

Supplement: S2 Fig — These are regions where induced mutations should not be called as they are more likely to be variation among Morex individuals. (TIFF) [file pgen.1011634.s002.tiff]

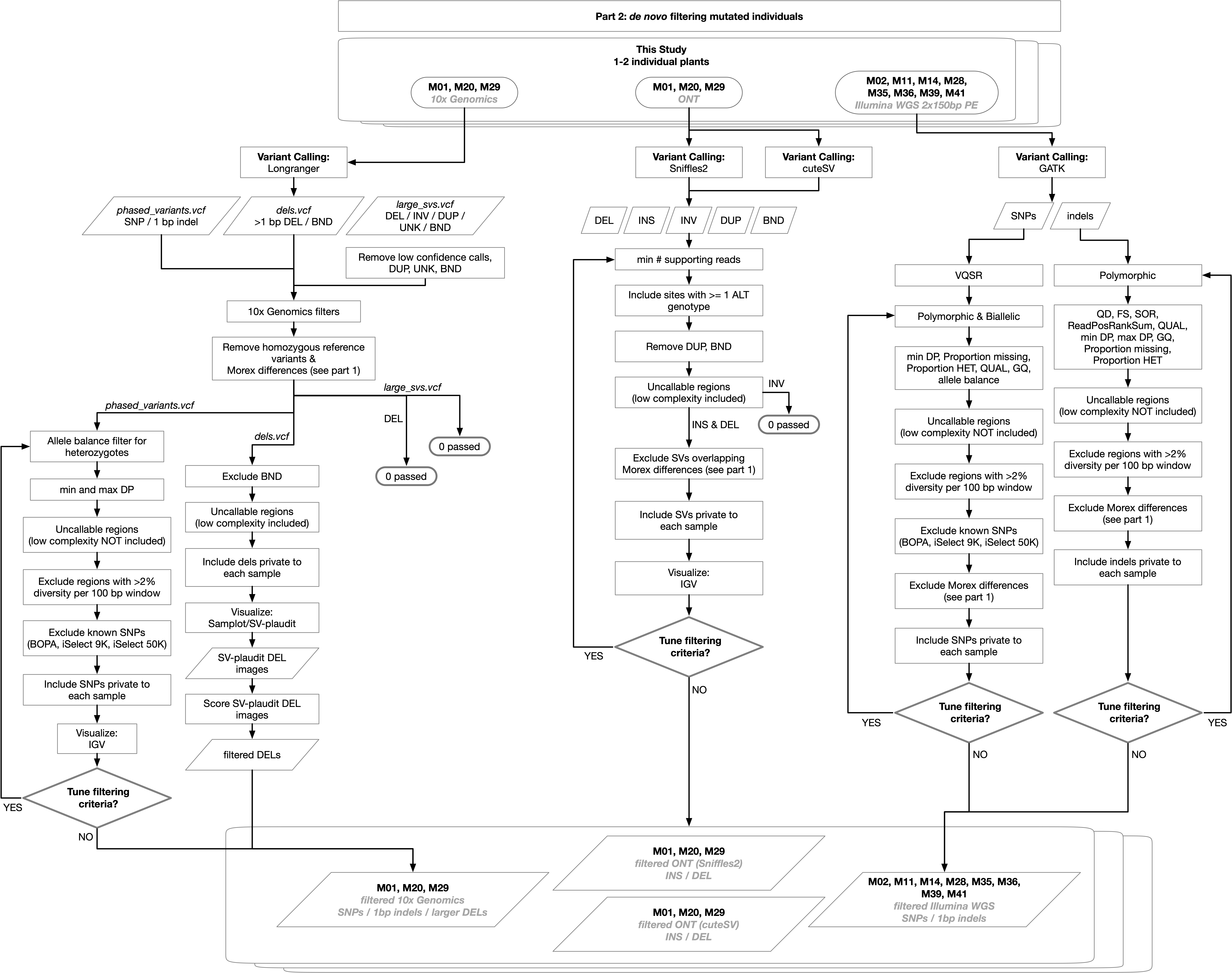

Supplement: S3 Fig — Larger SVs were visually evaluated with a similar approach as in part 1 of the filtering (S2 Fig). (TIFF) [file pgen.1011634.s003.tiff]

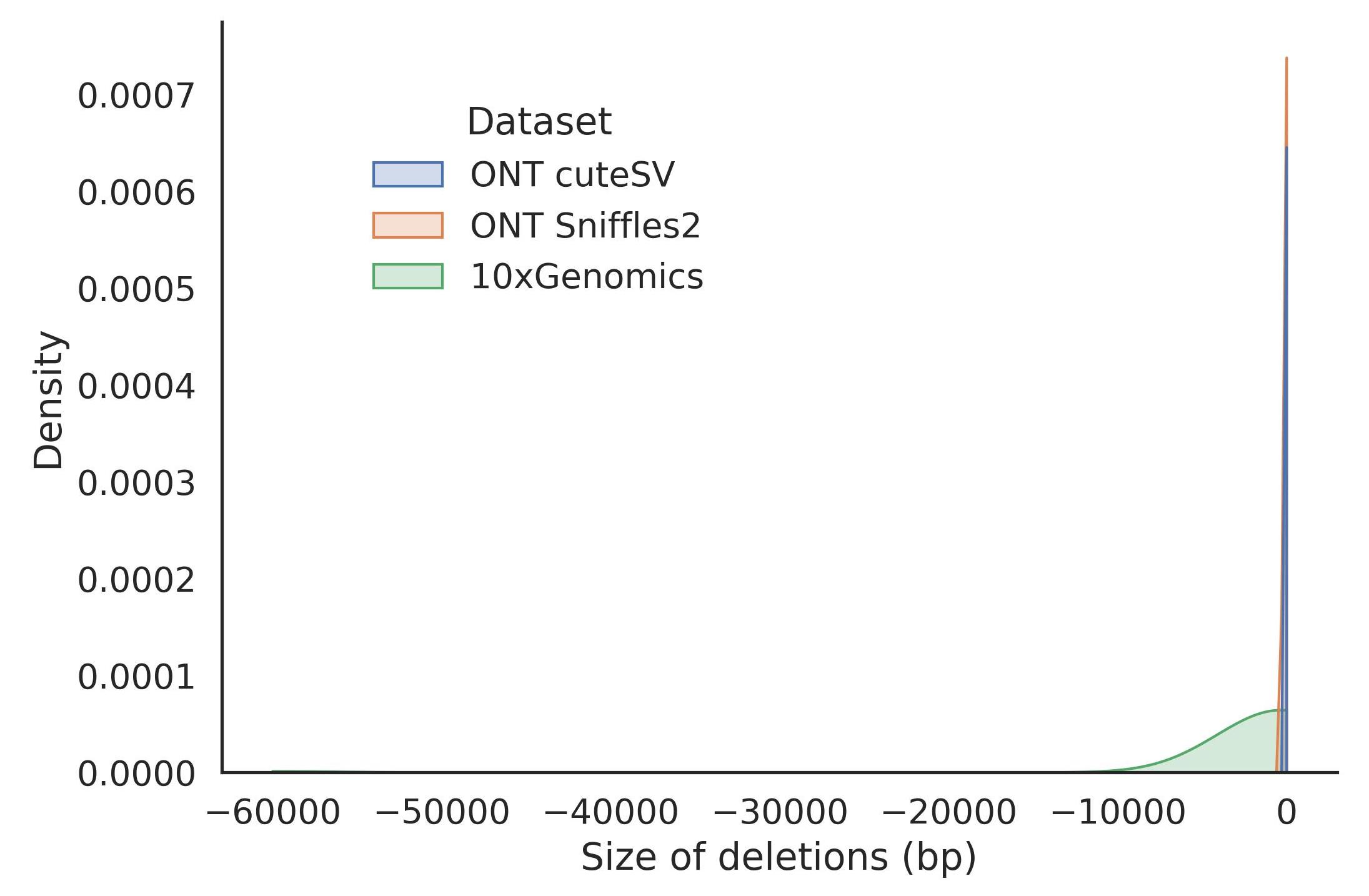

Supplement: S4 Fig — (TIFF) [file pgen.1011634.s004.tiff]

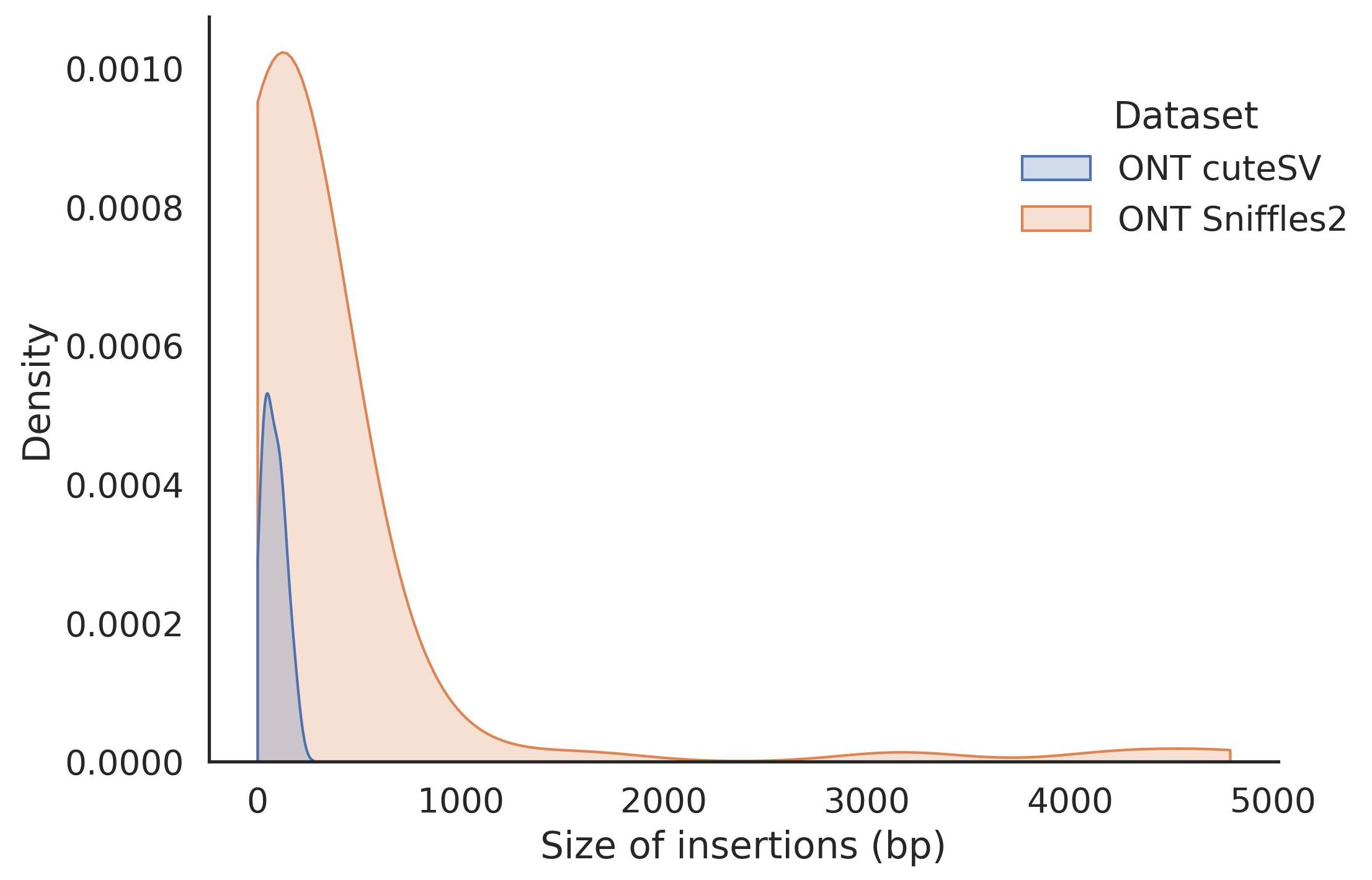

Supplement: S5 Fig — Larger insertions were not called in the 10X Genomics dataset. (TIFF) [file pgen.1011634.s005.tiff]

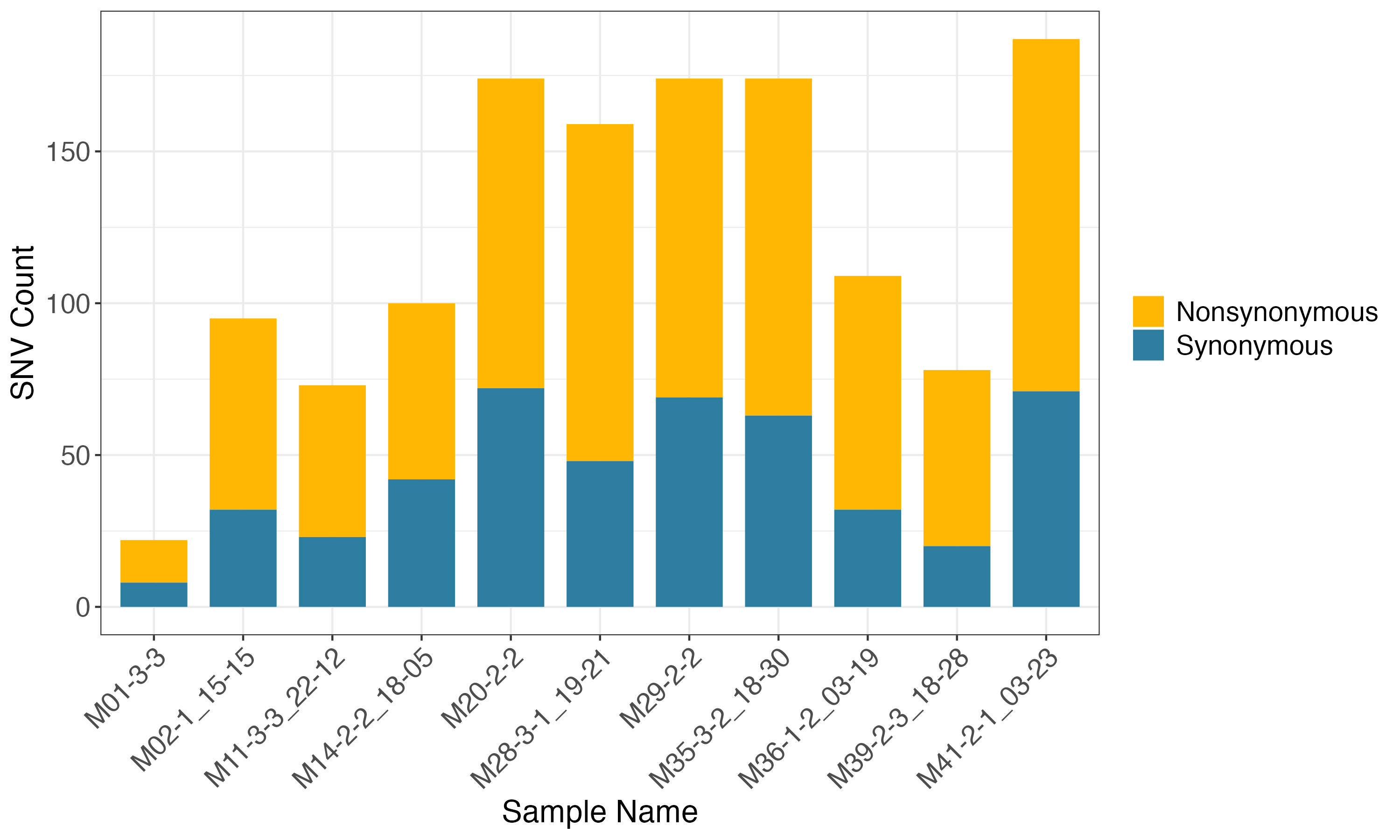

Supplement: S9 Fig — (TIFF) [file pgen.1011634.s009.tiff]

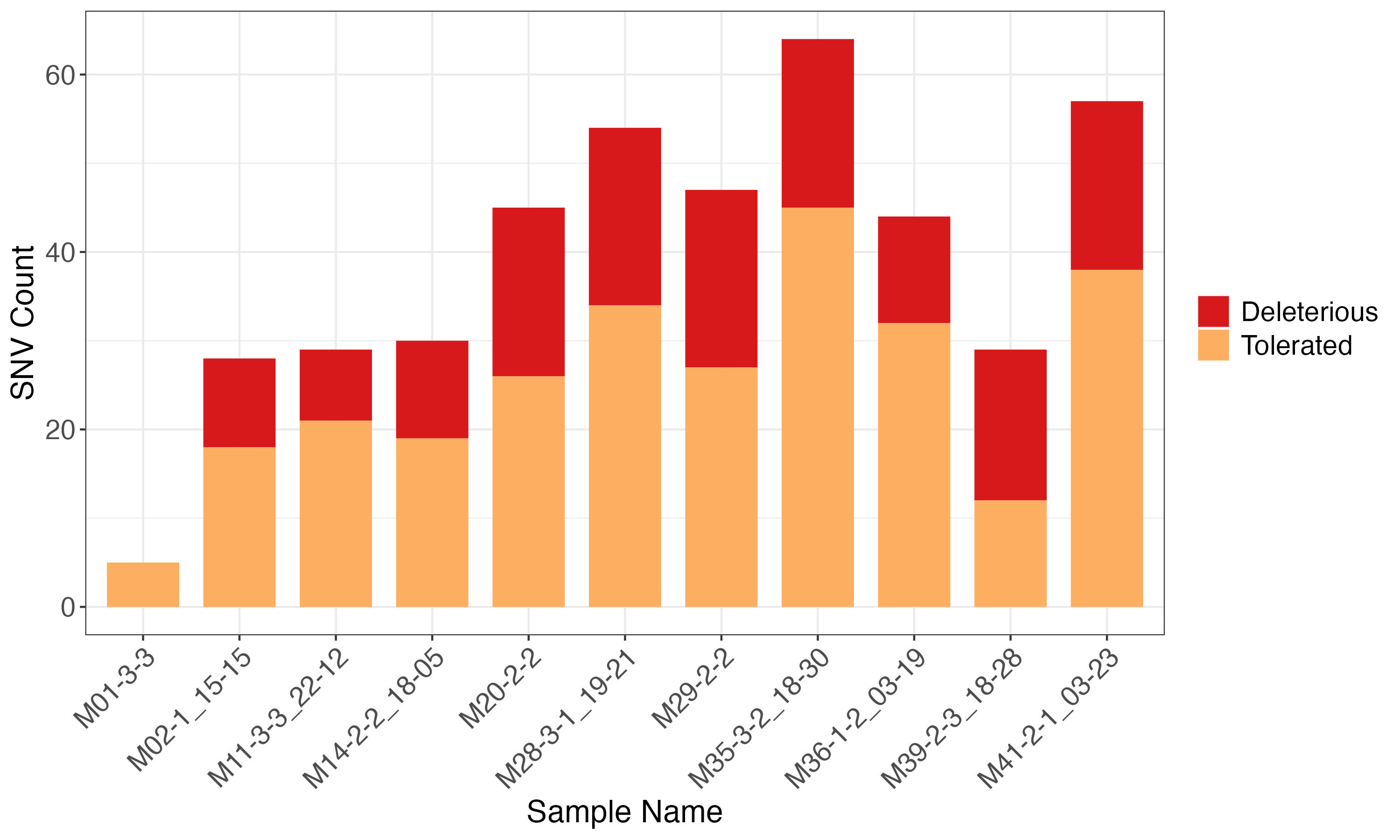

Supplement: S10 Fig — ”. (TIFF) [file pgen.1011634.s010.tiff]

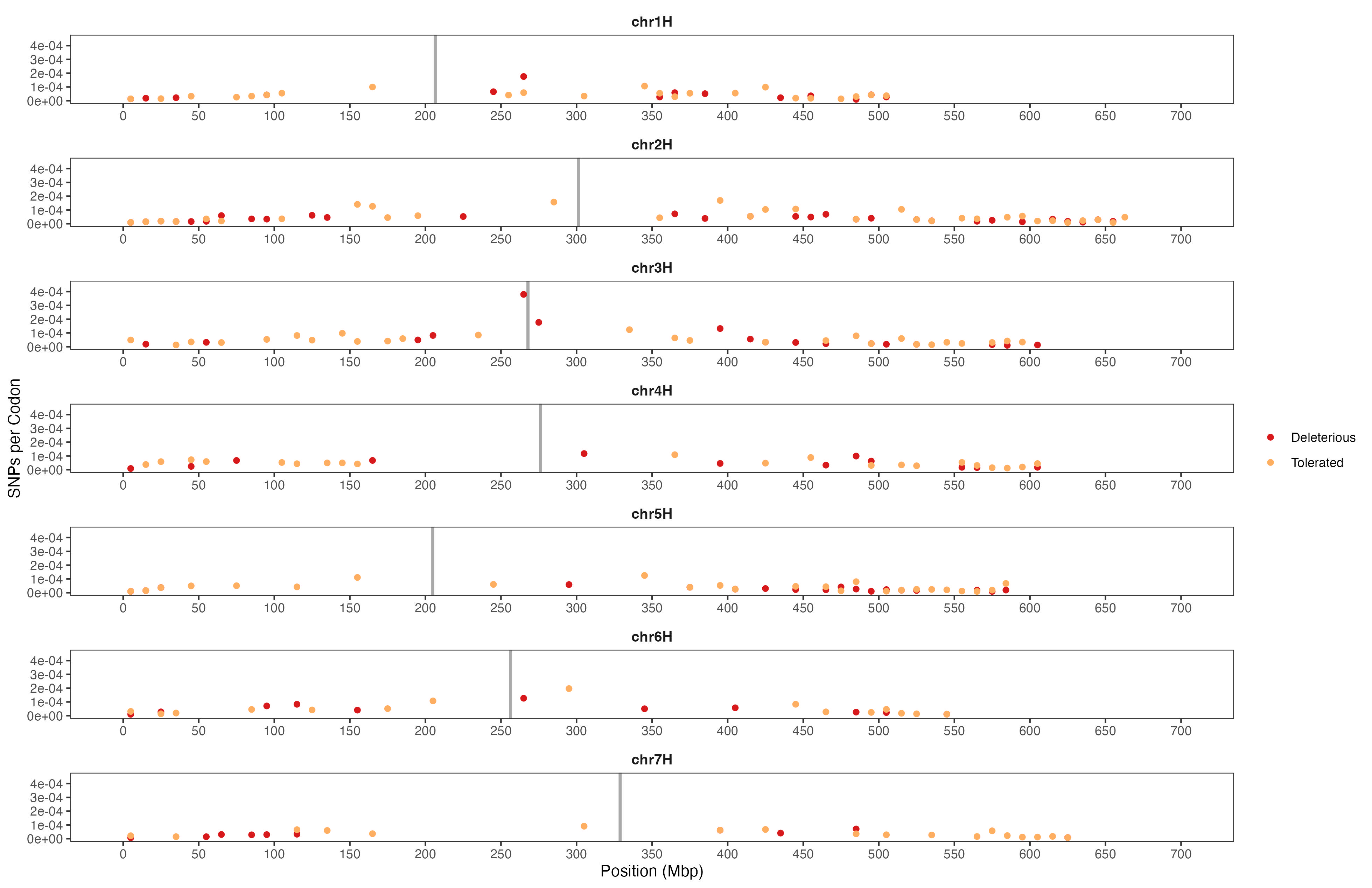

Supplement: S11 Fig — Nonsynonymous SNPs are separated into “Deleterious” vs. “Tolerated” and are plotted separately. The vertical grey line indicates the centromeric region. (TIFF) [file pgen.1011634.s011.tiff]

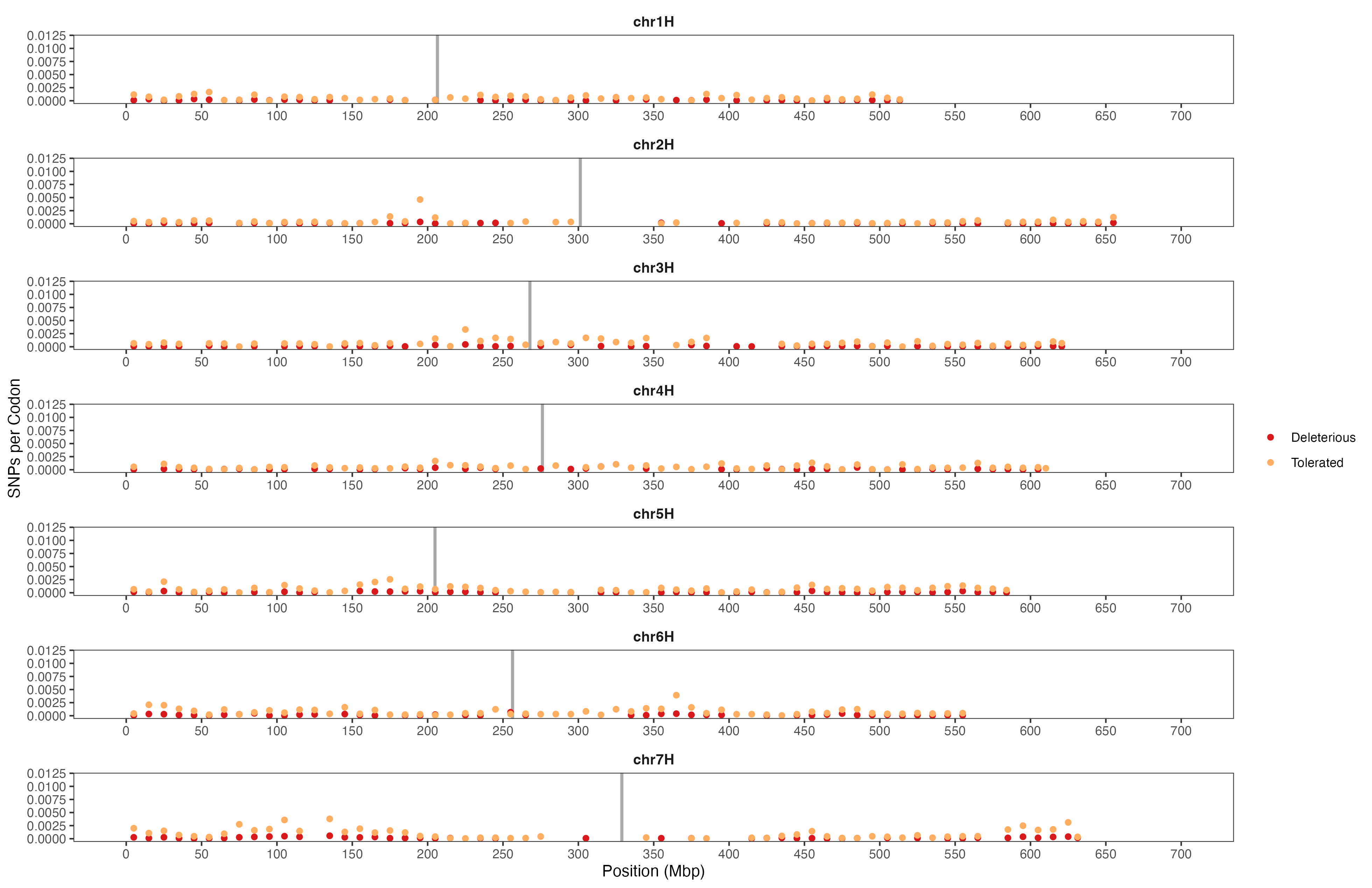

Supplement: S12 Fig — Nonsynonymous SNPs are separated into “Deleterious” vs. “Tolerated” and are plotted separately. The vertical grey line indicates the centromeric region. (TIFF) [file pgen.1011634.s012.tiff]

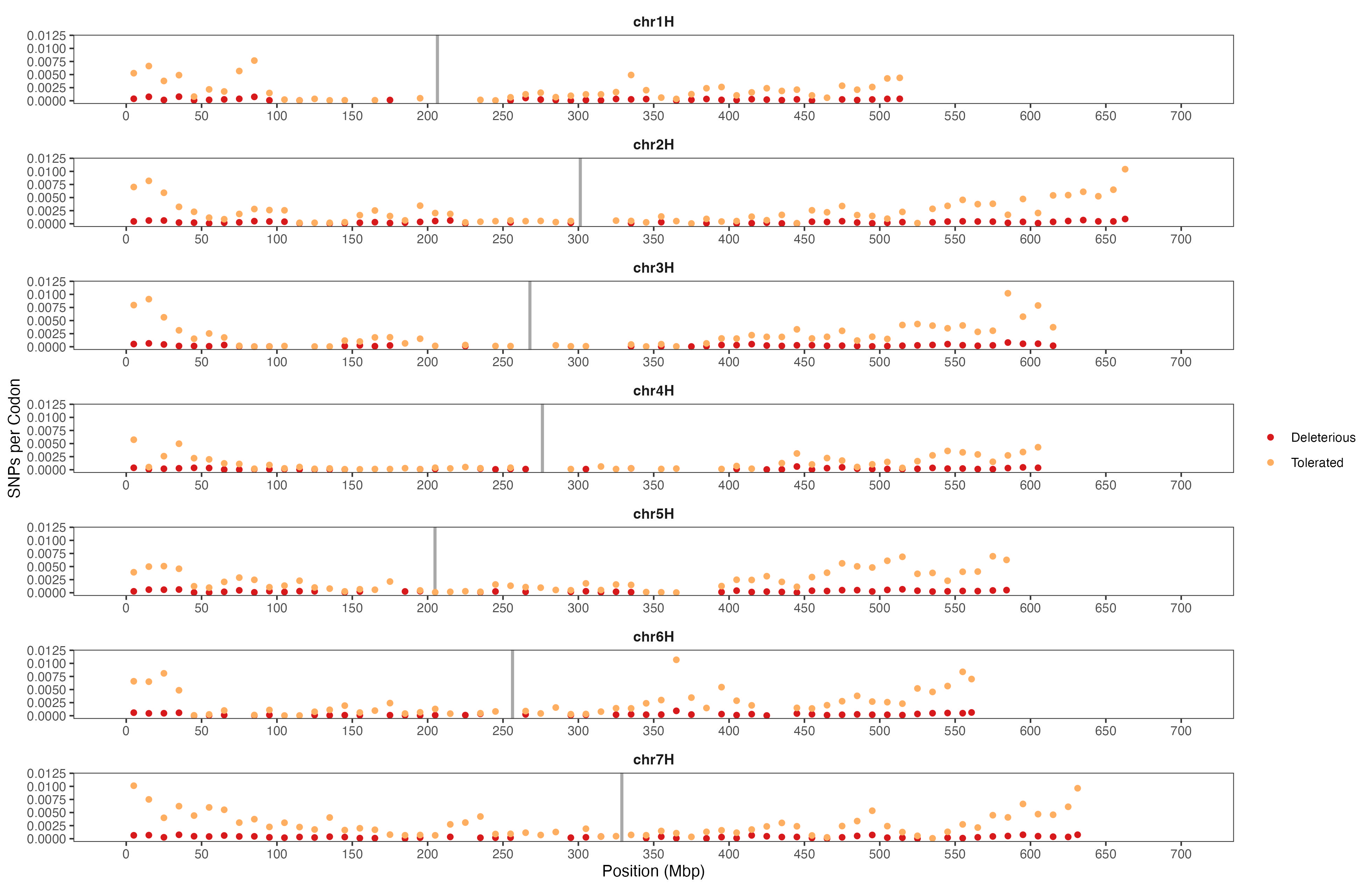

Supplement: S13 Fig — Nonsynonymous SNPs are separated into “Deleterious” vs. “Tolerated” and are plotted separately. The vertical grey line indicates the centromeric region. (TIFF) [file pgen.1011634.s013.tiff]

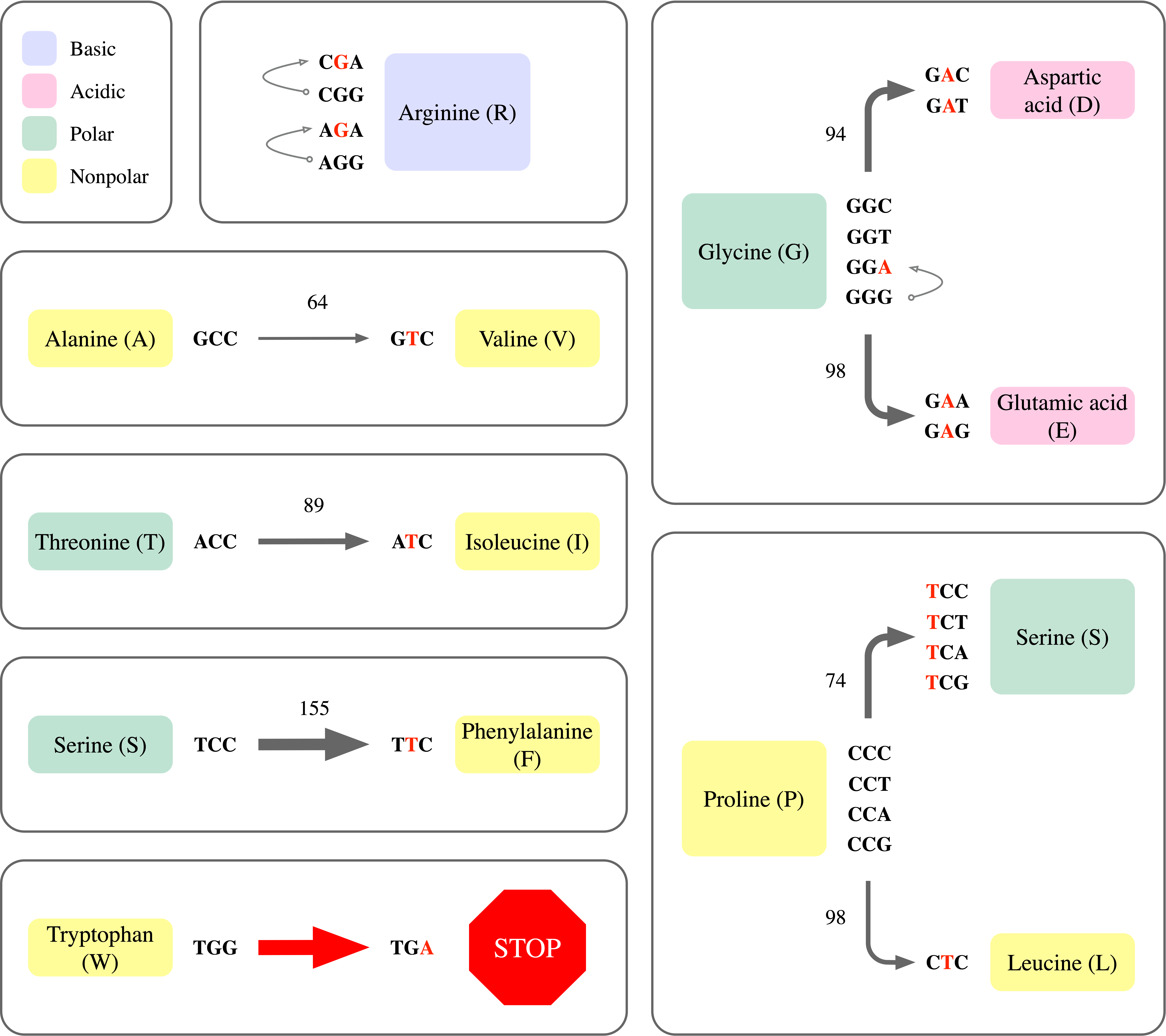

Supplement: S14 Fig — Colors represent the polarity of each amino acid. Arrows show the within codon changes, their line thickness, and the side numbers indicate the correspondent Grantham score. A thicker line indicates a greater evolutionary distance between two amino acids. Red letters represent the mutations induced by the sodium azide-associated motifs CC or reverse complement GG. Colors represent the primary properties of each amino acid, including polarity and acidity. (TIFF) [file pgen.1011634.s014.tiff]

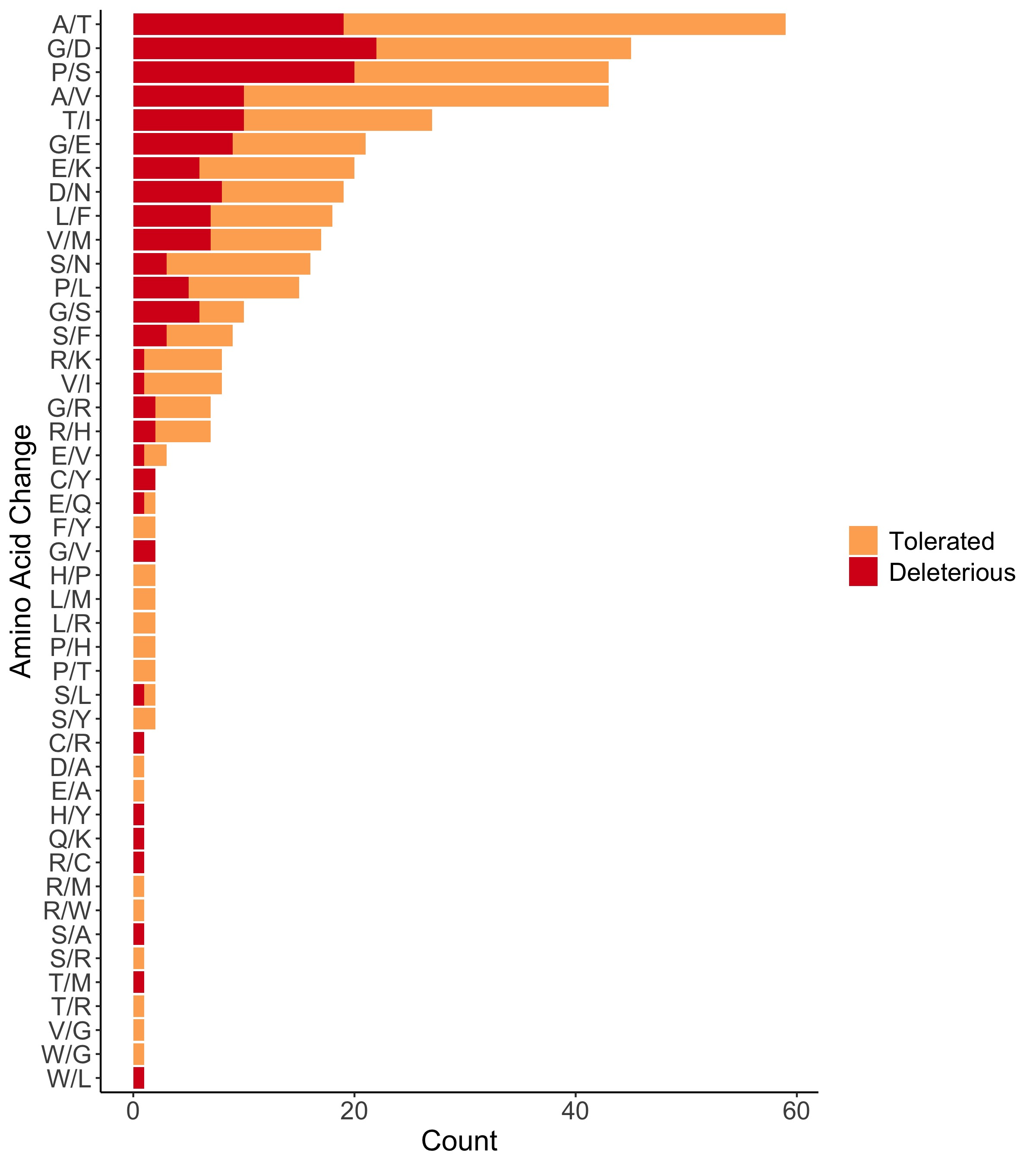

Supplement: S15 Fig — (TIFF) [file pgen.1011634.s015.tiff]

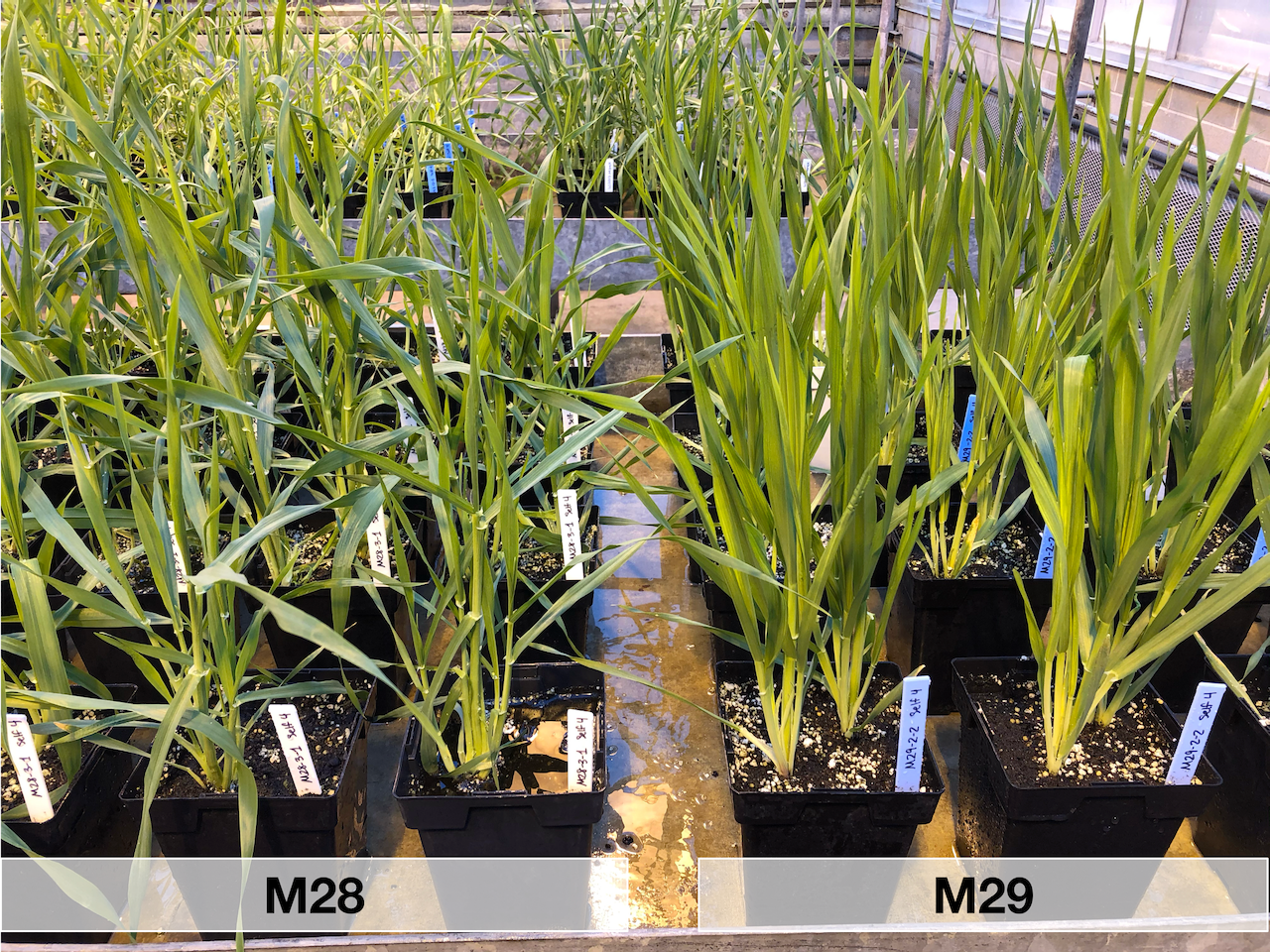

Supplement: S16 Fig — Both lines have been self-fertilized for four generations, and photos were taken five weeks after planting. (TIFF) [file pgen.1011634.s016.tiff]
