# Supplementary material for: Sodium azide mutagenesis induces a unique pattern of mutations
Source: PLoS Genet. 2025 Jun 3;21(6):e1011634. doi: 10.1371/journal.pgen.1011634 (PMC12162104; doi:10.1371/journal.pgen.1011634)
Supplement: Attachment — Submitted filename: comments.docx [file pgen.1011634.s021.docx]

The manuscript explores the genomic effects of sodium azide as a mutagen, presenting innovative findings and is recommended for acceptance. However, I have some questions regarding the paper:

1. The author describes the results of sodium azide mutagenesis throughout the article. Then, what is the specific difference between sodium azide mutagens and other mutagens in barley? Did not see the relevant content in the discussion.

2. The table S3 shows the correlation analysis data of Yield, Heading DAP, Height, but the original data of the phenotype of this correlation analysis are not found in the paper.

3. According to the Figure.5, the author selected 8 check lines. How did the author select these 8 lines? Why do we choose these 8 lines instead of others?

4. According to the Figure.5 and S17, Grain yield of 25 mutated lines, the Morex W2017 parent, and 8 check lines are different, but there is no relevant phenotypic picture to support it.

5. In this paper, the whole genome sequencing of 11 mutant lines, parents and 13 local lines was carried out. What are the differences between the phenotypic shapes of these 11 mutant lines and their parents? In addition, since the whole genome was sequenced, why only a broad-spectrum analysis of the sequencing was performed, then what specific changes in the genes between the selected 11 mutant lines and the parents, and whether the prediction of these genes function can construct potential validation phenotypic changes?

6. In Table 3, the harmful mutations do not show significant correlations with yield, heading days, and height. I hope the authors can discuss this result and whether similar conclusions have been drawn by other researchers.

7. Most sodium azide induced mutations occur in a specific nucleus sequence context, as C → t* changes in a CC mutation motif or the reverse completion This reflects the uniqueness of sodium azide. The article discusses the comparison with other studies, but it is not detailed enough. The comparison with other mutagens (such as EMS) and mutation patterns of other plants should be increased to highlight the uniqueness and advantages of sodium azide.
