# Supplementary material for: Sodium azide mutagenesis induces a unique pattern of mutations
Source: PLoS Genet. 2025 Jun 3;21(6):e1011634. doi: 10.1371/journal.pgen.1011634 (PMC12162104; doi:10.1371/journal.pgen.1011634)
Supplement: Attachment — Submitted filename: PGENETICS Reviewers 1 and 2 Comments.docx [file pgen.1011634.s023.docx]

**Reviewer #1**

The authors describe the analysis of 11 lines produced by Na azide mutagenesis. They distinguish induced mutations from the background providing spectrum, sequence context, and severity. The study appears fundamentally solid and is of interest because of the importance of induced mutations as a functional analysis and breeding tool, particularly with in the context of modern sequencing. Nevertheless, the manuscript requires several fixes, most in terms of clarity.

1. The manuscript language is often hard to follow, particularly in the results. The authors should make an effort to edit for clarity, stating the premises to each experiment or comparison, the expectations and the findings. I believe that each mutant line has 1000-4000 SNV, and 40 or 1500 ??? indels. Given the different methods for evaluating the indels, the results should specify what we can conclude from the analysis. For example, ~14 predicted severe mutants / line are observed. **What about the genes affected by the indels?** I think the information may be present, but it surely difficult to extract for a reader. I'm confused by the paragraph in Discussion ending in "This is a ~130-140 fold increase in the index mutation rate to an average of 5.13 x 10-7." What mutation rate? Similarly, I would like the results to make it clear where the following conclusion is coming from: "The typical line has an average of 273.3 disruptions of coding variants, including SNVs and indels."

Thanks, we appreciate the helpful feedback! We have edited the Result & Discussion and the Conclusions for clarity. With this submission, we have included a version of the manuscript with tracked changes, so it is relatively easy to see what has been modified.

Regarding genes affected by the indels, Figure S6 summarizes the number and proportion of indels that fall within and outside genes on a per-sample basis. We would be amenable to moving this figure to the main text, but it has a lot of numbers and is busy for a main figure.

We have clarified the source of results for the number of disruptions of coding variants – added Figure S6 call.

Regarding the question of the number of disruptive coding variants per line, we have updated the count to 249.1. This has been changed in the main text. We should note that the number now accurately reflects the values in Figure S6.

2. Figure 1 needs better explanation. For example, the untreated rare category seems to be referring to novel natural variants. However, I am not clear why some would occur in two lines and why there are so many of them. How many are estimated per generation? It seems that the lines are separated by ~ 5 generations (less than 10 in any case). How can 2M occur in such a short time if the previous standing variants (different from reference) are 4M? Further, all those that occurred in the last generation should be heterozygous (half of those in the previous, 1/4 of the one before, etc etc) and should provide an estimate of the per generation natural mutation.

   Thanks for pointing this out. The figure caption did need more explanation. We have added the following: “Private is defined as variants in mutated lines found in a single sample. Untreated are naturally occurring variants, with rare variants defined as those with a non-reference allele count ≤ 2, while untreated common variants have alternate allele count ≥ 3.

   With regard to why there are 2M variants, these are naturally occurring and representative of standing variation in cultivated barley. These variants have arisen over several hundred thousand generations since common ancestry, i.e., within the coalescent history of the species. We partition out rare (or rarer) variants because they are primarily newer mutations that should more closely reflect de novo mutations, whether spontaneously occurring or induced by the mutagen. A similar approach has been used in human resequencing studies, where the rarest class of variants is the closest proxy for new mutations (cf. Schaibley et al. 2014 - https://doi.org/10.1101/gr.154971.113).
3. Compared to EMS, this a relative low frequency of lesions. What I could not figure out, is how many mutations were observed to accumulate spontaneously per generation: 124 are predicted per line. Given the C->T predominance in the induced mutations compared to natural, it is clear that the authors are distinguishing the two categories. One criterion is that mutations are private (i.e. affect a single individual) while most natural variation is preexistent and common. New natural mutations, of course, would be private. **Could the numbers be provided per line to make this clear?** **Could the criteria for distinguishing induced from natural changes be made clearer?**

Thanks for this query. The reviewer is correct that induced variants are private to each mutated line. We have subtracted differences between our parent line “Morex-sample2” and the reference genome from the variants found in the mutated lines. Based on Table 2, this included 52,596 SNVs and 11,385 indels. There is a much more detailed account of how variants were filtered to get to de novo variants in each mutated line in the Materials and Methods under the header “Identifying de novo variants.” There is also a workflow for this process (admittedly very complicated), as depicted in Figure S2.

 The number of SNVs per line and the number of indels per line are depicted in Figure 1 (along with each mutant line name). This information is also listed in Table 2, which is broken into SNP and indel categories. We also indicate in both Figure 1 and Table 2 which lines were sequenced with 10X Genomics linked reads or Illumina PE; more indels are detected with 10X Genomics reads. We also depict per-sample variant information for nonsynonymous and synonymous SNVs in each mutated sample in Figure S9 and the number of putative deleterious and tolerated variants in each treated in Figure S10. See the spreadsheet identified as “NaN_spontaneous_mutation_calculations.xlsx” for detailed calculations.

Regarding criteria for distinguishing induced and natural (or spontaneous) mutations, we focus on the statistical patterns associated with changes in mutation frequency or the context of mutations. As noted within the manuscript, the mutations observed in the presence of a mutagen are necessarily a mixture of induced and natural changes (Zhu et al. 2020 – doi: 10.1534/genetics.120.303093).

4. Heterozygous for homozygous mutations. The selfing stage of the tested material predicts that most mutations and even more of the preexistent variation should be homozygous. The authors should present data addressing zygosity. I am not clear what filter was used to call a mutation. In other words, **what minimum coverage was used for homozygous and heterozygous calls**. The sequencing coverage has a large effect (Henry, 2014).

Homozygous and heterozygous variants were filtered on a per sample minimum depth > 5 (see Page 14). Additionally, heterozygous variants were filtered on allele balance (AB) deviation of 0.1 from the expected 0.5. AB is defined as alternate reads/(alternate reads + reference reads) (Pedersen et al. 2021 DOI: 10.1038/s41525-021-00227-3 and Muyas et al. 2018 DOI: 10.1002/humu.23674). These citations were added to the M&M section Page 14, L524-525. The details of filtering are in the GitHub repository for the project here: [https://github.com/MorrellLAB/Barley_Mutated/blob/master/01_snp_filtering](https://github.com/MorrellLAB/Barley_Mutated/blob/master/01_snp_filtering/)/.

5. Nomenclature of generations after mutagenesis. The authors seem to call the first generation produced directly from treated seed the M0 (See introduction: "Mutagenized plants are identified as the M0. "). On the other hand, Figure S1 could be interpreted to use M1 for the treated seed. Which applies? If the authors use M0, this is not a standard usage in barley, wheat, rice or arabidopsis and will generate confusion. This generation is commonly called the M1. See for example Gottwald, Barley, 2009; Hasegawa, Barley, 1984; Kleinhofs, Barley, 1978; Perry, Lotus, 2009; Green, Arabidopsis, 2003; Henry, Wheat, rice, 2014.

Thanks for pointing this out. We have worked with colleagues primarily involved in soybean research who use the M0 generation designator. We have updated Figure S1 nomenclature and image to “M1 plants” for clarity. The first generation produced from treated seed is the M1 in our study. Also, we have clarified the wording in the Material & Methods (Page 11).

6. The statement that the two round of selfing are needed to perform phenotypic screening is not supported by genetic theory or by the literature. The authors cite an FAO manual, which I could not access easily, but I doubt that it provides evidence toward this claim. Regardless, most screens are performed at the canonical M2 generation (one round of selfing), which already ensures that the mutations are meiotically inherited. One of course may elect to use the M3, but it is a matter of preference.

   Thanks for pointing out this issue. We have altered the wording in the main text to say that “Mutagenesis experiments often use multiple generations of self-fertilization to ensure that mutations are meiotically heritable (Nations 2018).” Our larger point in the paragraph was that the caution is required when screening for induced and other *de novo* mutations.

   For yield trials, the F3 generation is first generation which yields inbred plants derived from a unique set of F2 individuals subject to independent assortment and recombination events.
7. The issue of what LD50 is optimal is not easily determined. In many systems, chromosomal aberration contribute to the LD50 and the extent of genomic damage does not necessarily predict the point mutation density. The authors, in fact, provide direct evidence toward this because Na azide mutagenesis tends to induce fewer stop codons and splice site mutations than EMS. Therefore, the rate of lethal KO from mutagenesis is expected to be different for these two systems.

   We agree that calibrating an LD50 values is not easy. We made use of a published protocol that uses a specified concentration of sodium azide and a controlled pH (Döring et al. 1999 – doi: 10.1007/s004250050490). We mentioned the LD50 on line 68 primarily to point out that not all induced mutations will be observed.
8. The statement "This suggests that there are roughly 14 phenotype-changing variants per individual treated line." Should be rephrased because it is unlikely to be true for the following reasons: the majority of KO's in a diploid organism do not have a readily detected phenotype (see for example the experience with the homozygous mutant collection at Salk). Predicted deleterious mutations among missense ones have an even lower probability of phenotypic consequences. I think the author meant that 14 phenotypic changes are theoretically possible and consistent with the yield reduction observed. Indeed, only one mutant has a readily visible phenotype (i.e. not yield related).

We have reworded the sentence on page 7 to indicate that predicted deleterious SNVs have the potential to impact phenotypes but that the 14 phenotype-changing variants were not validated in a KO in this study. Note that Kono et al. 2018 - 10.1534/g3.118.200563 demonstrated using known phenotype-changing variants in *Arabidopsis thaliana* that nonsynonymous changes at highly conserved nucleotide sites are more likely to be phenotype-changing. However, many of these changes can be biochemical and are not directly observable phenotypes.

**Reviewer #2**

The manuscript explores the genomic effects of sodium azide as a mutagen, presenting innovative findings and is recommended for acceptance. However, I have some questions regarding the paper:

1. The author describes the results of sodium azide mutagenesis throughout the article. Then, what is the specific difference between sodium azide mutagens and other mutagens in barley? Did not see the relevant content in the discussion.

More than a half dozen mutagens (and multiple combinations of mutagens) have been used in barley, but much of this research occurred in the 1960s-1970s (enumerated in Lundqvist & Lunqvist 1988, doi: 10.1186/s41065-023-00304-w). Most of the screening of mutants has focused on their phenotypic effects. We have added a citation to Hansson et al. 2024 “A guide to barley mutants” (doi: 10.1186/s41065-023-00304-w). We cited both studies that reported the nucleotide sequence-based effects of mutagens in barley (Olsen et al. 1993; Knudsen et al. 2022).

2. The table S3 shows the correlation analysis data of Yield, Heading DAP, Height, but the original data of the phenotype of this correlation analysis are not found in the paper.

The raw and spatially adjusted data for the phenotypes have been added to the Data Repository University of Minnesota (DRUM repository, akin to Figshare) (see files “[mmx 2020-2022_trial avg_031623.csv](https://www.dropbox.com/scl/fi/q3e2oibmdszqu6j1mobxs/mmx-2020-2022_trial-avg_031623.csv?rlkey=ky9twqwm2h6hab1a03cu29rkl&st=pnctw6jc&dl=0)” and “[mmx 2020-2022_plot level_031623.csv](https://www.dropbox.com/scl/fi/k1kn4mtz5d9v6hx2sg5qz/mmx-2020-2022_plot-level_031623.csv?rlkey=v1y6hwy3u9q5gkydrw9ghbyh5&st=22mmi09f&dl=0)”). Yield values across a yield trial are difficult to visualize, but we have included plots of raw and spatially adjusted yield trials in the GitHub repository associated with a previous, related study. These depictions were not represented in the prior publication because of their functional nature (cf. https://github.com/MorrellLAB/Deleterious_GP/tree/master/Results/Phenotypic_Plots).

3. According to the Figure.5, the author selected 8 check lines. How did the author select these 8 lines? Why do we choose these 8 lines instead of others?

Page 12 - A standard set of check lines is used to compare yield and disease resistance in new barley crosses and breeding lines. The checks used here are chosen as appropriate for yield testing in spring, 6-row malting barley lines grown at multiple locations in the Upper Midwest of the United States. The checks are chosen because of known yield and performance characteristics.

4. According to the Figure.5 and S17, Grain yield of 25 mutated lines, the Morex W2017 parent, and 8 check lines are different, but there is no relevant phenotypic picture to support it.

Raw yield data was collected for each year of the trial and spatially adjusted based on a moving average (see Tiede & Smith 2018 - doi: 10.1007/s11032-018-0820-3; Kono et al. 2019 – doi: 10.1534/genetics.119.302733). Phenotypic data were spatially adjusted with a moving average across the field plots. As noted above, images of raw and spatially adjusted yield data using the same approach (and some of the same field locations are available here: <https://github.com/MorrellLAB/Deleterious_GP/tree/master/Results/Phenotypic_Plots>).

In addition, below are pictures of the lowest yielding line (M36) compared to W2017 Morex (the parent of the mutated lines) and Lacey (the check line).

5. In this paper, the whole genome sequencing of 11 mutant lines, parents and 13 local lines was carried out. What are the differences between the phenotypic shapes of these 11 mutant lines and their parents? In addition, since the whole genome was sequenced, why only a broad-spectrum analysis of the sequencing was performed, then what specific changes in the genes between the selected 11 mutant lines and the parents, and whether the prediction of these genes function can construct potential validation phenotypic changes?

Ten of the 11 mutated lines show no notable phenotypic change from the Morex parental line. A goal of this project was to create lines that carry de novo mutations that would cause a diminution in fitness but not be so disruptive as to be irrelevant for barley breeding and production lines. This is intended to emulate the types of variants that occur as standing deleterious variants. Thus, there are no noteworthy phenotypes outside of M29, where the phenotype is quite distinct from typically barley lines (see the photograph included here as Figure S16).

6. In Table 3, the harmful mutations do not show significant correlations with yield, heading days, and height. I hope the authors can discuss this result and whether similar conclusions have been drawn by other researchers.

Previous examinations of mutational load in cultivated barley have found a reduction in mutational load (the count of derived harmful mutations) over multiple generations of selection (Kono et al. 2019, doi: 10.1534/genetics.119.302733). However, traits not directly related to yield (through effects on plant health and fitness) are not expected to be directly impacted by harmful mutations. For Kono et al. 2019 this meant limited effect of deleterious mutations on the levels of a fungal disease-related compound or plant height were not observed.

More broadly, this is similar to the conundrum of "yield" QTL in general. There are many small effect genes for a trait with lower heritability. It is possible that a comparison of variant effects on yield component traits (like seed weight) might identify stronger effects. Seed weight is more heritable, and thus, it is possible that a specific variant may have a larger effect. (We don’t have seed weight values for each replicate of the experiment.)

7. Most sodium azide induced mutations occur in a specific nucleus sequence context, as C → T* changes in a CC mutation motif or the reverse completion This reflects the uniqueness of sodium azide. The article discusses the comparison with other studies, but it is not detailed enough. The comparison with other mutagens (such as EMS) and mutation patterns of other plants should be increased to highlight the uniqueness and advantages of sodium azide.

The nucleotide sequence-level characterization of the nature of induced mutations and the sequence context in which they occur is limited. The most directly comparable studies (that focus on the nature of the nucleotide change) are two studies of NMU-induced mutations in mice and a few previous studies from Luca Comai and colleagues. We cited some but not all of these studies, but we have now fully explored this contrast. We have added citations to the Conclusions, which identify three additional papers discussing the phenotypic effects of barley mutagenesis and exploring the nucleotide sequence-level effects of EMS, NMU, etc.

---

**Additional changes:**

1. NCBI SRA BioProject numbers added to Table 1
